# Complete Pancreatic Agenesis Presenting With Neonatal Diabetes and Exocrine Insufficiency: A Case Report

**DOI:** 10.7759/cureus.96536

**Published:** 2025-11-11

**Authors:** Faisal Alshareef, Abdullah H Alfares, Dalal Alshathri

**Affiliations:** 1 Pediatrics, Security Forces Hospital, Riyadh, SAU; 2 Information Science, King Saud University, Riyadh, SAU

**Keywords:** exocrine pancreatic insufficiency, hemorrhagic stroke, neonatal diabetes, pancreatic agenesis, znf808 mutation

## Abstract

Pancreatic agenesis is one of the most challenging genetic conditions to manage. This case report describes a male infant with complete pancreatic agenesis due to a homozygous *ZNF808* mutation, presenting with permanent neonatal diabetes mellitus (PNDM) and exocrine pancreatic insufficiency (EPI). Born at 38 weeks with severe intrauterine growth restriction (IUGR), the patient developed insulin-dependent diabetes by three months of age, with undetectable C-peptide levels and absent pancreatic tissue on imaging. His clinical course was complicated by influenza-associated acute respiratory distress syndrome (ARDS), resulting in hemorrhagic stroke and post-stroke epilepsy, along with chronic lung disease and global developmental delay. Management required a multidisciplinary approach, including basal-bolus insulin therapy, pancreatic enzyme replacement, nutritional optimization, antiepileptic drugs, and respiratory support. This case underscores the severe complications associated with this condition and illustrates the critical need for a comprehensive management strategy.

## Introduction

Congenital pancreatic agenesis is an exceptionally rare developmental disorder characterized by the complete absence of pancreatic tissue, resulting in permanent neonatal diabetes mellitus (PNDM) and exocrine pancreatic insufficiency (EPI). First described in the medical literature in the 1960s, fewer than 50 confirmed cases have been reported worldwide, making it one of the most severe forms of pancreatic dysgenesis [1]. The condition arises from disruptions in pancreatic embryogenesis, typically due to mutations in critical transcription factors governing pancreatic development, such as *PDX1*, *PTF1A*, *GATA6*, and, more recently, *ZNF808* [2]. Clinically, affected infants present shortly after birth or in early infancy with severe hyperglycemia requiring insulin therapy, along with malabsorption and failure to thrive due to absent digestive enzymes. Without prompt diagnosis and multidisciplinary management, the condition carries significant morbidity and mortality risks, including diabetic ketoacidosis (DKA), severe malnutrition, and neurodevelopmental impairment [3].

The genetic basis of pancreatic agenesis has been increasingly elucidated over the past two decades. Early studies identified *PDX1* (pancreatic and duodenal homeobox 1) as a key regulator of pancreatic development, with biallelic mutations leading to pancreatic agenesis in both humans and mouse models [4]. Subsequent research revealed that *PTF1A* and *GATA6* mutations could also cause pancreatic agenesis, often in association with additional congenital anomalies such as cardiac defects, biliary atresia, or cerebellar hypoplasia [5]. More recently, whole-exome sequencing has uncovered novel genetic causes, including ZNF808, a zinc-finger protein whose exact role in pancreatic development is still under investigation [6]. These discoveries have refined diagnostic algorithms for neonatal diabetes, emphasizing the importance of genetic testing in cases with concurrent exocrine insufficiency [7].

Despite advances in genetic diagnosis, the management of pancreatic agenesis remains challenging due to the lifelong need for insulin and pancreatic enzyme replacement, as well as the high risk of complications such as recurrent infections, metabolic instability, and developmental delays. Previous case reports highlight the variability in outcomes, with some patients surviving into adulthood with careful management, while others succumb to metabolic crises or severe infections in early childhood [8].

## Case presentation

The patient, a male infant, was born at 38 weeks of gestation via Caesarean section to a 24-year-old mother. The primary indication for delivery was placental insufficiency leading to severe, late-onset asymmetrical intrauterine growth restriction (IUGR). His birth weight was 1.3 kg, well below the third percentile for gestational age. The Apgar scores were fair at seven and nine at one and five minutes, respectively. Due to low birth weight and feeding intolerance, he was admitted to the neonatal intensive care unit (NICU). He underwent standard glucose monitoring for severe IUGR during the first 72 hours, with all values within the normal range (45-100 mg/dL). During his 28-day NICU stay, he exhibited significant difficulty establishing oral feeds, necessitating gavage feeding and meticulous caloric supplementation. No evidence of major neonatal sepsis, significant cholestasis, or respiratory distress was observed. He was discharged on day of life 28 with a weight of 1.8 kg and on partial oral feeding (meaning he required supplemental gavage feeds to meet his full caloric needs). His weight gain trajectory remained suboptimal at approximately 15-18 grams per day, attributed at the time to the sequelae of severe IUGR and generalized weakness.

Early infancy and diabetes onset

At approximately three months of age, routine follow-up revealed incidental mild hyperglycemia. The infant was clinically asymptomatic, with no signs of dehydration or ketoacidosis. Capillary blood glucose measurements ranged between 180 and 200 mg/dL (10-11 mmol/L), and repeat testing confirmed persistent hyperglycemia. There was no history of high-dose steroid exposure or other stressors that could account for transient hyperglycemia, raising suspicion for neonatal diabetes mellitus. Further biochemical evaluation demonstrated undetectable C-peptide levels, indicating negligible endogenous insulin production. By four months of age, a definitive diagnosis of permanent neonatal diabetes mellitus was established. Insulin therapy was initiated using a basal-bolus regimen, consisting of long-acting insulin degludec (Tresiba) for basal coverage and rapid-acting insulin aspart for prandial glucose management. Given the infant’s small size and variable feeding patterns, insulin dosing was carefully titrated to mitigate hypoglycemic risk.

During this period, the parents reported intermittent episodes of abnormal "jerky" movements and staring spells. Initial concern for hypoglycemic seizures prompted glucose monitoring during these events, which yielded normal values.

Neurologic complications

At five months of age, the patient developed high fever and cough, leading to a diagnosis of influenza A (H1N1) pneumonia. His respiratory status rapidly deteriorated, necessitating hospitalization and progression to acute respiratory distress syndrome (ARDS), which required mechanical ventilation in the pediatric intensive care unit (PICU). During this critical illness, he experienced an acute episode of diminished responsiveness accompanied by right-sided body twitching. An emergent cranial CT scan revealed a left intraparenchymal hemorrhage. The immediate workup for the stroke revealed significant coagulopathy (platelet count 58x10⁹/L, D-dimer >5.0 µg/mL) and concurrent systolic hypertension (readings of 110-125 mmHg) during the peak of his systemic illness, supporting the attribution of the hemorrhage to ARDS-induced coagulopathy and hypertension. Following intensive management, the infant survived the ARDS.

In the subsequent weeks, he developed recurrent seizures. Electroencephalogram (EEG) demonstrated epileptiform discharges localized to the left hemisphere. Given the identical clinical semiology and the new confirmation of a structural brain lesion from the stroke, the earlier abnormal movements were reclassified as the initial presentation of his epilepsy, for which the stroke provided a definitive structural cause. Antiepileptic therapy was initiated, achieving good seizure control.

Diagnosis of pancreatic agenesis

The constellation of neonatal-onset diabetes, persistent malabsorption (evidenced by steatorrhea), and failure to thrive prompted suspicion of a congenital pancreatic defect. At seven months of age, an abdominal ultrasound failed to visualize pancreatic tissue, raising the initial suspicion. A subsequent contrast-enhanced abdominal CT confirmed complete pancreatic agenesis, with no identifiable pancreatic head, body, or tail. Instead, loops of bowel occupied the expected pancreatic location - a radiographic hallmark known as the "dependent stomach sign". Concurrent stool analysis revealed severe exocrine pancreatic insufficiency, with fecal elastase levels <50 µg/g (normal >200 µg/g).

Given parental consanguinity (first-cousin marriage), an autosomal recessive genetic disorder was suspected. Whole-exome sequencing identified a homozygous loss-of-function mutation in the *ZNF808 *gene. This molecular diagnosis confirmed autosomal recessive pancreatic agenesis type 3 (PAGEN3).

He experienced two episodes of DKA at six and nine months of age, both triggered by intercurrent infections. Each episode presented with dehydration, metabolic acidosis, and hyperglycemia (>500 mg/dL), necessitating intravenous insulin and fluid resuscitation.

Abdominal imaging incidentally detected multiple tiny hepatic calcifications, possibly residual from a subclinical, transient neonatal cholestasis. Renal ultrasound revealed right renal hypoplasia (3.0 cm, >2 SD below the mean for age, placing it below the fifth percentile) compared to the normal left kidney (5.5 cm). Renal function was normal based on serum creatinine (0.3 mg/dL), blood urea nitrogen (BUN) (15 mg/dL), and urinalysis.

Following ARDS, he required prolonged oxygen support, suggestive of chronic lung disease. At 10 months, he was discharged on low-flow nasal cannula oxygen (0.5-1 L/min), which remained necessary at 12 months.

By 12 months, he exhibited significant global developmental delay, failing to meet expected gross motor or language milestones. This was attributed to a multifactorial etiology, including extreme IUGR, critical illness burden, and neurological injury from intracranial hemorrhage.

Laboratory investigations

As illustrated in Table 1, initial newborn metabolic screening results were within normal limits. At three months of age, when hyperglycemia was first detected, laboratory evaluation demonstrated consistently elevated random blood glucose levels of 11 mmol/L (approximately 200 mg/dL) on two separate measurements. Blood gas analysis revealed a normal pH of 7.37 without evidence of ketonemia, effectively ruling out diabetic ketoacidosis at initial presentation. Hemoglobin A1c measured at four months of age was elevated at 6.8% (normal range 4%-5.5% for infants), reflecting chronic mild hyperglycemia. Endocrine evaluation showed profoundly low serum insulin levels (<2 μU/mL) with correspondingly low C-peptide levels, confirming absent pancreatic beta-cell function. Pancreatic autoantibodies (including anti-insulin, glutamic acid decarboxylase (GAD), and islet cell antigen (IA-2) antibodies) were negative, supporting a non-autoimmune etiology. Evaluation of exocrine function revealed severe pancreatic insufficiency with fecal elastase <50 μg/g (normal >200 μg/g). Stool microscopy demonstrated fat globules and a positive Sudan stain, confirming steatorrhea secondary to fat malabsorption. Nutritional assessment identified mild deficiencies of fat-soluble vitamins D and E, prompting initiation of supplementation. During subsequent episodes of diabetic ketoacidosis, laboratory findings included severe hyperglycemia (>500 mg/dL or 27 mmol/L), metabolic acidosis (pH ~7.05) with elevated anion gap, and detectable serum ketones, all of which resolved with appropriate treatment.

**Table 1:** Summary of Laboratory Investigations DKA: Diabetic ketoacidosis.

Test	Patient's Finding	Normal Range	Timing / Context
Random Blood Glucose	11 mmol/L (≈200 mg/dL)	~3.9-5.6 mmol/L (70-100 mg/dL) (Fasting) [1]	3 months of age
Venous pH	7.37	7.35-7.45	Initial presentation (3 months)
Hemoglobin A1c (HbA1c)	6.8%	4-5.5% (for infants) [2]	4 months of age
Serum Insulin	<2 μU/mL	3-20 μU/mL (fasting) [3]	Post-diagnosis
Serum C-peptide	Low / Undetectable	0.9-4.3 ng/mL (fasting) [3]	Post-diagnosis
Pancreatic Autoantibodies	Negative (Anti-insulin, GAD, IA-2)	Negative	Post-diagnosis
Fecal Elastase	<50 μg/g	>200 μg/g [4]	7 months of age
Stool Sudan Stain	Positive (fat globules)	Negative	Post-diagnosis
Vitamin D	Deficient	Sufficient >20 ng/mL (50 nmol/L) [5]	Nutritional assessment
Vitamin E	Deficient	Varies by age and assay	Nutritional assessment
Blood Glucose (during DKA)	>27 mmol/L (>500 mg/dL)	3.9-5.6 mmol/L (70-100 mg/dL) [1]	Episodes at 6 and 9 months
Venous pH (during DKA)	~7.05	7.35-7.45	Episodes at 6 and 9 months
Serum Ketones	Detectable/Elevated	Negative/Trace	Episodes at 6 and 9 months
Anion Gap	Elevated	8-16 mEq/L [6]	Episodes at 6 and 9 months

Imaging studies

**Figure 1:**
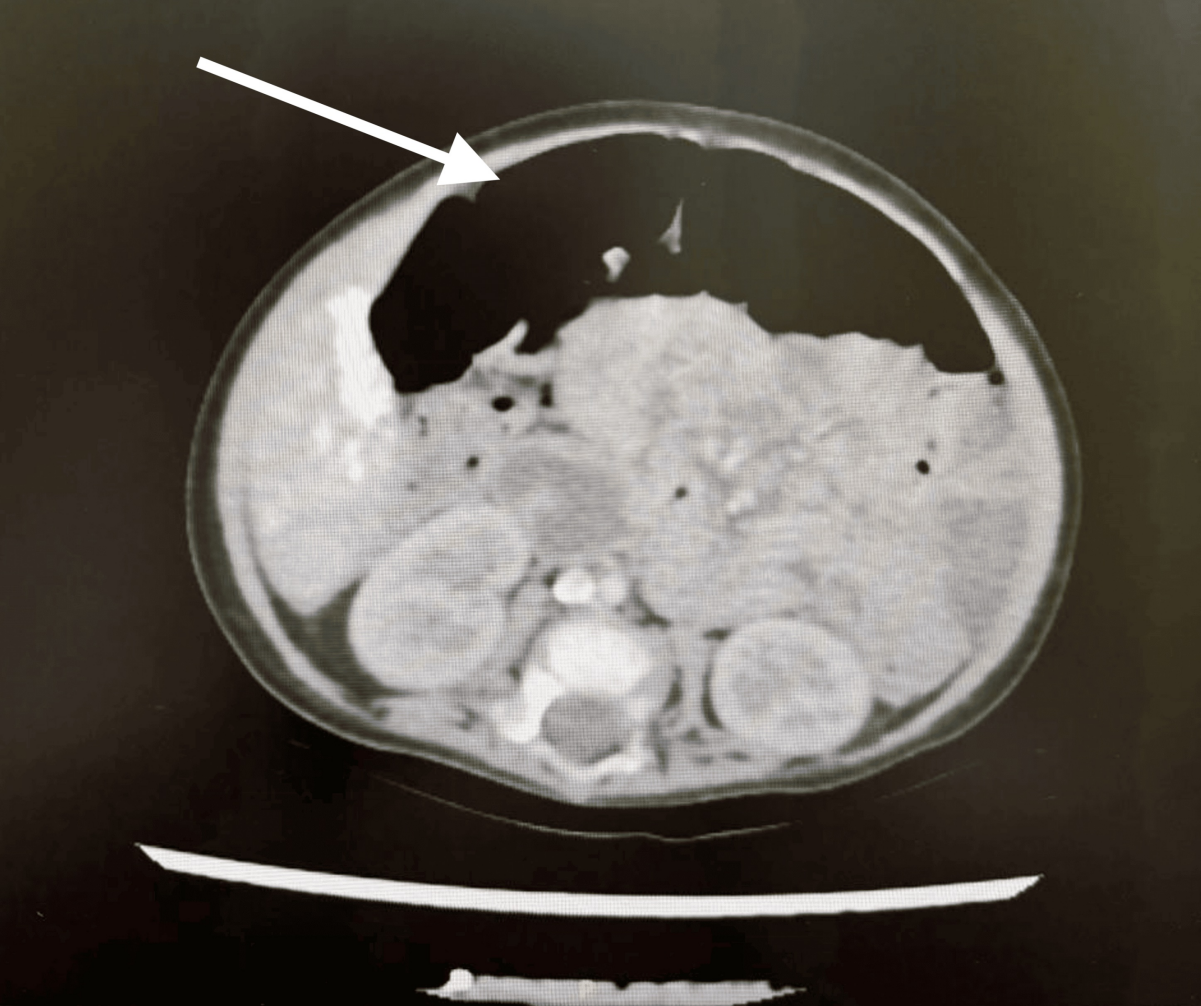
Abdominal CT scan obtained at seven months of age demonstrated complete pancreatic agenesis

Diagnostic imaging played a crucial role in establishing the diagnosis. The definitive abdominal CT scan obtained at seven months of age demonstrated complete pancreatic agenesis, with no identifiable pancreatic tissue in the expected retroperitoneal location (Figure 1). The characteristic findings included close approximation of the duodenum and stomach due to absent pancreatic tissue, along with medial malrotation of the spleen. The study also revealed multiple small hepatic calcifications without hepatosplenomegaly. Neuroimaging with brain MRI at six months of age showed left parietotemporal encephalomalacia corresponding to the site of previous intraparenchymal hemorrhage, with surrounding gliosis but no hydrocephalus. Notably, neonatal cranial ultrasounds obtained during the NICU admission had been normal, confirming the postnatal nature of the brain injury. Serial chest radiographs during the acute phase of ARDS demonstrated bilateral diffuse infiltrates, while follow-up imaging at one year of age revealed residual mild pulmonary fibrosis. Renal ultrasound identified right renal hypoplasia (3.0 cm, below the fifth percentile) compared to the normal left kidney (5.5 cm), without evidence of hydronephrosis. Echocardiography showed normal cardiac structure and function, ruling out associated congenital heart defects.

Genetic testing

Following radiographic confirmation of pancreatic agenesis, a comprehensive genetic evaluation was pursued. Initial targeted sequencing of neonatal diabetes-associated genes (including *KCNJ11*, *ABCC8*, *INS*, and the *6q24* locus) yielded negative results. Whole-exome sequencing identified a homozygous frameshift mutation in the *ZNF808* gene (chromosome 19q13.41), with both parents confirmed as heterozygous carriers, consistent with autosomal recessive inheritance. This molecular finding established the diagnosis of pancreatic agenesis type 3 (PAGEN3), as previously described in the literature. No pathogenic variants were detected in other known pancreatic agenesis-associated genes (*PDX1*, *PTF1A*). The identified *ZNF808* mutation has been documented in multiple unrelated cases of isolated pancreatic agenesis, supporting its pathogenicity. Genetic counseling was provided to the family, including a discussion of the 25% recurrence risk for future pregnancies.

Diagnosis

The comprehensive diagnostic evaluation confirmed congenital pancreatic agenesis as the underlying etiology of the patient's complex presentation. The final diagnosis included: permanent neonatal diabetes mellitus secondary to absent insulin-producing beta cells; severe exocrine pancreatic insufficiency due to complete absence of acinar tissue; secondary neurological complications including epileptic encephalopathy following post-influenza intracerebral hemorrhage; chronic lung disease consequent to ARDS; congenital right renal hypoplasia; and global developmental delay attributable to combined effects of extreme IUGR, critical illness, and neurological injury.

The differential diagnosis initially considered other causes of neonatal diabetes, including transient neonatal diabetes mellitus (*6q24* imprinting abnormalities) and monogenic forms of diabetes (*KCNJ11*/*ABCC8* mutations). However, the persistence of hyperglycemia beyond the neonatal period and requirement for ongoing insulin therapy argued against transient neonatal diabetes. The presence of exocrine pancreatic insufficiency and fat malabsorption distinguished this case from isolated monogenic diabetes. While cystic fibrosis was considered given its association with pancreatic insufficiency and diabetes, the absence of characteristic clinical features and negative newborn screening made this diagnosis unlikely. The constellation of radiographic findings demonstrating complete pancreatic agenesis, coupled with the genetic identification of a pathogenic *ZNF808* mutation, provided definitive confirmation of congenital pancreatic agenesis as the unifying diagnosis.

Management

The management of this complex case required a coordinated multidisciplinary approach addressing the patient's endocrine, gastrointestinal, neurological, respiratory, and developmental needs. Given the rarity and severity of pancreatic agenesis with its multisystem complications, treatment was carefully tailored to optimize growth, metabolic control, and neurodevelopmental outcomes.

Lifelong insulin replacement therapy was initiated using a basal-bolus regimen appropriate for infancy. Basal coverage was achieved with once-daily insulin degludec (Tresiba), while rapid-acting insulin aspart was administered pre-prandially to address postprandial glycemic excursions. Given the patient's small size and variable nutritional intake, insulin doses were meticulously adjusted based on frequent capillary blood glucose monitoring. Continuous glucose monitoring (CGM) was implemented as soon as technically feasible, providing valuable trend data and early detection of asymptomatic hypoglycemia. Target glucose levels were maintained between 100 and 200 mg/dL (5.5-11 mmol/L), balancing glycemic control with hypoglycemia risk. During intercurrent illnesses, insulin requirements were proactively increased with more frequent monitoring to prevent diabetic ketoacidosis.

Pancreatic enzyme replacement therapy with enteric-coated pancrelipase microspheres was initiated at a dose of approximately 2,000 units of lipase per kilogram per feeding, consistent with established pediatric guidelines for EPI (e.g., Cystic Fibrosis Foundation consensus guidelines). Caregivers were trained in proper administration techniques, including opening capsules and mixing contents with acidic foods to ensure enzymatic activity. Nutritional management focused on high-calorie feedings supplemented with medium-chain triglycerides (MCT) oil to bypass the need for pancreatic lipase. Fat-soluble vitamin (A, D, E, K) supplementation was provided at supranormal doses due to impaired absorption, with particular attention to correcting initial vitamin D insufficiency. Vitamin B12 supplementation was added prophylactically given the potential for impaired absorption from protein-bound sources. Despite these interventions, growth parameters remained at approximately the fifth percentile, reflecting the ongoing challenges of pancreatic insufficiency.

The patient's epilepsy was managed with a combination of levetiracetam (Keppra) administered twice daily and nocturnal clonazepam for breakthrough seizures. Phenobarbital, initially used during the acute phase, was successfully weaned by 12 months of age due to concerns about potential neurodevelopmental effects. The current antiepileptic regimen has maintained good seizure control for three consecutive months. Given the extent of cortical injury evident on MRI, long-term anticonvulsant therapy is anticipated.

Chronic lung disease secondary to ARDS necessitated ongoing oxygen supplementation via nasal cannula at 0.5-1 L/min to maintain oxygen saturations above 94%. Pulmonary management included the transient use of diuretics for bronchopulmonary dysplasia-like symptoms and palivizumab prophylaxis against respiratory syncytial virus (RSV) infection. Respiratory support was complemented by chest physiotherapy and bronchodilators as needed. At one year of age, the patient continued to require supplemental oxygen during sleep and feeds, though gradual weaning is anticipated as lung tissue continues to mature.

A robust early intervention program was implemented to address global developmental delays. This included physical therapy for gross motor skills, occupational therapy focusing on feeding and fine motor development, and speech therapy emphasizing oral-motor skills. Care coordination was achieved through a multidisciplinary clinic involving endocrinology, gastroenterology, neurology, pulmonology, and developmental pediatrics specialists. The family received ongoing psychosocial support and genetic counseling to address the implications of the autosomal recessive condition.

Given the patient's history of severe influenza virus infection and ongoing respiratory compromise, particular attention was paid to infection prevention. This included strict adherence to recommended immunization schedules with emphasis on annual influenza vaccination. A low threshold was maintained for evaluating and treating potential infections, given the patient's increased vulnerability to metabolic decompensation during illness.

## Discussion

This case presents a rare and complex presentation of complete pancreatic agenesis, confirmed by imaging and genetic testing as PAGEN3 due to a homozygous *ZNF808 *mutation. The patient’s clinical course highlights the multisystem challenges associated with congenital pancreatic absence, including PNDM, EPI, and subsequent neurological, respiratory, and developmental complications. This discussion contextualizes the case within the existing literature, emphasizing diagnostic approaches, management strategies, and long-term outcomes in similar reported cases.

Complete pancreatic agenesis is an extremely rare condition, with fewer than 50 cases reported in the literature. Most cases are attributed to mutations in critical pancreatic developmental genes, including *PDX1*, *PTF1A*, *GATA6*, and, more recently, *ZNF808* [9,10]. The present case aligns with the findings of De Franco et al. (2021), who identified *ZNF808* as a novel cause of isolated pancreatic agenesis in multiple consanguineous families [9]. Unlike *PDX1* or *GATA6* mutations, which are often associated with additional congenital anomalies (e.g., cardiac defects or cerebellar hypoplasia), *ZNF808*-related agenesis appears to predominantly affect the pancreas, though extra-pancreatic features (e.g., renal hypoplasia in this case) may still occur [11].

The patient’s presentation with severe IUGR and early-onset diabetes (three months) mirrors previous reports of pancreatic agenesis, where insulin deficiency manifests shortly after birth or in early infancy [12]. Notably, the absence of C-peptide and pancreatic autoantibodies helped differentiate this condition from transient neonatal diabetes or autoimmune diabetes. The concurrent exocrine insufficiency, evidenced by undetectable fecal elastase and steatorrhea, further supported the diagnosis, as seen in other cases of pancreatic agenesis [13].

A striking feature of this case was the development of a hemorrhagic stroke following influenza-associated ARDS, leading to post-stroke epilepsy. While neurological injury is not a classic feature of pancreatic agenesis, severe metabolic instability (e.g., recurrent diabetic ketoacidosis) and critical illness likely contributed to this complication. Similar cases of PNDM with neurological sequelae have been reported, particularly in *KCNJ11* or *ABCC8* mutations, where epilepsy may arise from direct channelopathy effects [14]. 

The management of epilepsy in this context required a careful selection of anticonvulsants to avoid exacerbating metabolic or developmental risks. Levetiracetam was chosen due to its favorable safety profile in infants, as supported by studies demonstrating minimal cognitive impact compared to phenobarbital [15]. The addition of clonazepam for nocturnal seizures follows recommendations for focal epilepsy in pediatric patients with structural brain lesions [16].

The patient’s chronic lung disease post-ARDS underscores the vulnerability of infants with pancreatic agenesis to respiratory complications. Similar cases of severe viral pneumonia progressing to ARDS have been reported in PNDM patients, possibly due to impaired immune responses or metabolic stress [17]. The requirement for long-term oxygen supplementation aligns with studies showing that infants with ARDS, particularly those with preexisting growth restriction, are at high risk for persistent pulmonary abnormalities [18].

Nutritional management in pancreatic agenesis remains challenging due to combined insulin deficiency and malabsorption. The use of pancreatic enzyme replacement therapy (PERT) and MCT oil-based feeds is well-documented in EPI, though optimal dosing in infants is not standardized [19]. This patient’s suboptimal weight gain (fifth percentile) despite aggressive nutritional support reflects findings from a 2020 cohort study, where pancreatic agenesis patients often remained below growth norms despite therapy [20].

Long-term outcomes in pancreatic agenesis are poorly defined due to disease rarity. However, existing reports suggest high morbidity related to metabolic instability, infections, and neurodevelopmental delays [21]. A 2019 review of *PDX1*-related agenesis cases found that 60% of patients required lifelong insulin and enzyme therapy, with developmental delays noted in 40% [22]. The present case’s global developmental delay likely results from multifactorial insults (IUGR, stroke, critical illness), emphasizing the need for early intervention.

## Conclusions

This case illustrates the significant multisystem effects of *ZNF808*-related pancreatic agenesis and emphasizes the necessity of early genetic testing in infants with neonatal diabetes and EPI. Several key takeaways emerge from this case. First, obtaining a genetic diagnosis is vital for determining prognosis and guiding family counseling, especially given the 25% recurrence risk in autosomal recessive inheritance. Second, a coordinated multidisciplinary approach is essential to address the complex endocrine, gastrointestinal, neurological, respiratory, and developmental needs of these patients. Third, proactive infection prevention is critical due to the high risk of severe metabolic complications during intercurrent illnesses.

Future research should focus on optimizing PERT dosing for infants and exploring targeted treatments for *ZNF808*-related pathology. Furthermore, ensuring long-term neurodevelopmental follow-up is crucial for improving outcomes in this vulnerable population.
